# Trends in use of acupuncture among adults in Taiwan from 2002 to 2011: A nationwide population-based study

**DOI:** 10.1371/journal.pone.0195490

**Published:** 2018-04-10

**Authors:** Mei-Yao Wu, Yu-Chen Lee, Cheng-Li Lin, Ming-Cheng Huang, Mao-Feng Sun, Hung-Rong Yen

**Affiliations:** 1 Department of Chinese Medicine, China Medical University Hospital, Taichung, Taiwan; 2 Graduate Institute of Acupuncture Science, College of Chinese Medicine, China Medical University, Taichung, Taiwan; 3 Management Office for Health Data, China Medical University Hospital, Taichung, Taiwan; 4 Graduate Institute of Chinese Medicine, School of Chinese Medicine, College of Chinese Medicine, China Medical University, Taichung, Taiwan; 5 Research Center for Traditional Chinese Medicine, Department of Medical Research, China Medical University Hospital, Taichung, Taiwan; 6 Research Center for Chinese Herbal Medicine, China Medical University, Taichung, Taiwan; 7 Department of Biotechnology, Asia University, Taichung, Taiwan; 8 Chinese Medicine Research Center, China Medical University, Taichung, Taiwan; Stanford University School of Medicine, UNITED STATES

## Abstract

In recent years, acupuncture has gained in popularity worldwide. However, recent epidemiological studies are lacking. We conducted this study to investigate the trends in acupuncture utilization among adults in Taiwan from 2002 to 2011. We analyzed data from the Longitudinal Health Insurance Database 2000 (LHID 2000), which contains all original claims data for 1 million beneficiaries randomly sampled from the registry of all beneficiaries enrolled in the National Health Insurance (NHI) program in 2000. The one-year prevalence of acupuncture use among adults increased from 7.98% in 2002 to 10.9% in 2011. Acupuncture use significantly increased yearly (incidence rate ratio = 1.04, 95% CI = 1.03−1.05, p<0.001). Patients who were female, were middle-aged, resided in highly urbanized areas and suffered from injury or disorders of the musculoskeletal system were prone to more frequent acupuncture use. Our study revealed that the utilization of acupuncture became increasingly popular in Taiwan from 2002 to 2011. Our findings may provide useful information for clinical practice and research as well as for health policy decision making.

## Introduction

An increasing number of patients worldwide have become interested in complementary and alternative medicine (CAM) in recent years [1]. Of the different types of CAM, traditional Chinese medicine (TCM) has been well defined as an ancient medical system by the National Center for Complementary and Integrative Health (NCCIH, U.S.A.) [2]. The trends in TCM utilization have also increased gradually in Taiwan, and more than 28% of Taiwanese consulted a TCM service in 2010 [3].

Acupuncture, one of the treatment approaches in TCM, has been widely used in Asian [4–6] and Western countries [7–10]. It has been included in the clinical practice guidelines for the treatment of pain-related diseases [11, 12]. For example, the American College of Physicians and the American Pain Society recommended acupuncture as a non-pharmacological treatment method for low back pain [13]. Acupuncture has been practiced not only in local clinics but also in most teaching hospitals and medical centers in Taiwan [5]. In a previous Taiwanese study, more than 6% of subjects had received acupuncture in 2002 [5].

The National Health Insurance (NHI) program was established in 1995 in Taiwan, and more than 99% of the population were enrolled in the NHI program by the end of 2010 [3]. The NHI program covers not only Western medical services but also ambulatory care in TCM. All claims data from the NHI program are collected in the National Health Insurance Research Database (NHIRD), and researchers can use these datasets to evaluate the utilization of TCM for various diseases such as allergies [14, 15], musculoskeletal diseases [16–18], metabolic disorders [19, 20], gynecologic diseases [21], pediatric diseases [2], geriatric disorders [22] and cancer [23, 24]. De-identified demographic characteristics (e.g., sex, date of birth, occupation and place of residence) and clinical information (e.g., diagnosis, management and treatment) are also provided in the database. This nationwide database is highly reliable, which reduces the potential for sampling bias [25].

To date, only a few studies have addressed questions about the trends in utilization of acupuncture. Some studies have only reported acupuncture usage in specific diseases [26], whereas others were conducted decades ago. For example, a previous study described the demographics and patterns of acupuncture use in Taiwan from 1996 to 2002 [5]. To understand the trends in acupuncture use in Taiwan, we conducted this nationwide population-based study to investigate the utilization of acupuncture from 2002 to 2011.

## Materials and methods

### Data source

We accessed the Longitudinal Health Insurance Database 2000 (LHID2000) from the National Health Research Institutes, Taiwan. The LHID2000 contains all of the original claims data for 1 million beneficiaries who were randomly sampled from the registry of all beneficiaries enrolled in the NHI program in 2000, and these randomly sampled 1 million individuals were followed longitudinally through 2011 according to their personal identification numbers. The included individuals were removed from the cohort until death or withdrawal from the NHI program. The beneficiary characteristics, including age, geographic region, and place of residence, are updated each year. De-identified demographic data on sex, date of birth, residence and occupation as well as medical records of clinical visits, hospitalizations, diagnosis codes and treatment codes were all included in the datasets.

### Study samples

We analyzed adult acupuncture users by the treatment codes, which included manual acupuncture (B41, B42, B80-B84, B90-B94, P27041, P31103, P32103 and P33031), electroacupuncture (B43, B44, B86-89 and P33032) and complex acupuncture (B45 and B46). The NHI defines “complex acupuncture” as acupuncture treatment for patients with specific disorders, such as cerebral vascular disease, spinal cord injury, cancer and psychiatric disorders, which require more effort to treat. The diagnosis codes in the LHD2000 were consistent with the *International Classification of Diseases*, *Ninth Revision*, *Clinical Modification* (ICD-9-CM) [27].

### Study variables

Sex, age, level of urbanization and geographic region were chosen as independent variables to explore their effects on acupuncture utilization. Of the 1 million beneficiaries, adults older than 20 years were included in this study. Acupuncture users were categorized into 7 sub-groups according to age: 20 to 29, 30 to 39, 40 to 49, 50 to 59, 60 to 69, 70 to 79 and ≥80 years old. There were 6 geographic regions assessed, including Taipei and the Northern, Central, Southern, Kao-Ping and Eastern regions of Taiwan. Residence areas were grouped into 4 levels of urbanization based on population density (people/km^2^), the ratio of elderly persons, the ratio of people with different educational levels, the ratio of agricultural workers and the number of physicians per 100,000 persons [28]. The highest degree of urbanization was level 1, and the lowest was level 4.

### Statistical analysis

The demographic characteristics and medical record data were analyzed by SAS statistical software (version 9.4 for Windows; SAS Institute, Inc., Cary, NC, USA). The data analysis comprised descriptive statistics of the demographic characteristics of acupuncture users. The percentage of acupuncture users by different demographic factors was calculated as the number of acupuncture users dividing by the sampled enrollees by different demographic factors. Logistic regression analysis was used to investigate the change in the utilization rate of acupuncture over time. Generalized estimating equation (GEE) method was used to determine the statistically significant of the trend of acupuncture usage in the follow-up period.

### Ethical consideration

All names and identification numbers of enrollees and names of medical facilities included in the NHIRD dataset for this study were encrypted using a random alphanumeric series to protect the privacy of the subjects and adhere to ethical considerations. None of the research members could identify any enrollee or facility from the dataset (https://nhird.nhri.org.tw/en/). This study was approved by the Research Ethics Committee of China Medical University and Hospital (CMUH104-REC2-115) and the National Health Research Institutes, which maintain and manage the NHI database.

## Results

Adults older than 20 years who were included in the 1 million beneficiaries of the LHID2000 dataset were included in this study. The valid beneficiaries were 677,7542 in 2002 and 732,466 in 2011 (Table 1). The proportion of acupuncture users increased significantly from 7.98% in 2002 to 10.9% in 2011. The analysis of generalized estimating equation revealed that acupuncture use increased significantly with year (incidence rate ratio = 1.04, 95% CI = 1.03−1.05, p<0.0001, data not shown). The number of total acupuncture visits in one year increased from 132,522 in 2002 to 176,538 in 2011. There was no significant difference in the average visit times of acupuncture users from 2.45 in 2002 to 2.22 in 2011.

**Table 1:** Number of patients and visits among acupuncture users from 2002–2011 in Taiwan.

Year	Valid beneficiaries	Acupuncture users								
		Total number (%)	Female (%)	Male (%)	Urbanization				New subjects (%)	Total visits
					Level 1	Level 2	Level 3	Level 4		
2002	677752	54106 (7.98)	30136 (8.96)	23970 (7.02)	18535 (8.92)	15830 (7.97)	9893 (7.98)	9848 (6.68)	35944 (66.4)	132522
2003	683220	55967 (8.19)	31256 (9.20)	24711 (7.19)	18928 (9.03)	16527 (8.24)	10321 (8.26)	10191 (6.88)	30334 (54.2)	142635
2004	688815	58120 (8.44)	32866 (9.59)	25254 (7.30)	19315 (9.14)	17319 (8.55)	10765 (8.55)	10721 (7.19)	27388 (47.1)	139478
2005	697144	55768 (8.00)	31296 (9.01)	24472 (7.00)	18373 (8.60)	16624 (8.10)	10378 (8.13)	10393 (6.90)	22450 (40.3)	131372
2006	703958	55069 (7.82)	30839 (8.78)	24230 (6.87)	18664 (8.65)	16337 (7.88)	9926 (7.68)	10142 (6.68)	20014 (36.3)	121376
2007	710048	57253 (8.06)	32302 (9.10)	24951 (7.03)	19407 (8.93)	16965 (8.10)	10271 (7.87)	10610 (6.94)	19079 (33.3)	124680
2008	716391	60529 (8.45)	34143 (9.52)	26386 (7.38)	20463 (9.34)	18022 (8.52)	11005 (8.35)	11039 (7.18)	18942 (31.3)	133124
2009	721504	71416 (9.90)	40310 (11.1)	31106 (8.65)	24177 (11.0)	21340 (10.0)	13113 (9.86)	12786 (8.27)	21878 (30.6)	159299
2010	727666	78735 (10.8)	43859 (12.0)	34876 (9.63)	26973 (12.1)	23370 (10.9)	14881 (11.1)	13511 (8.68)	22909 (29.1)	171313
2011	732446	79536 (10.9)	44362 (12.0)	35174 (9.66)	27220 (12.2)	23465 (10.8)	14981 (11.0)	13870 (8.86)	20246 (25.5)	176538

Total numbers of acupuncture users means total numbers of different acupuncture users in each year.

% of female (male) acupuncture users means the percentage of female acupuncture users in female (male) beneficiaries in each year.

% of acupuncture users in the different urbanization levels means the percentage of acupuncture users in the beneficiaries of each urbanized area.

Our data revealed that female acupuncture users increased from 8.96% in 2002 to 12.0% in 2011, where male acupuncture users increased from 7.02% in 2002 to 9.66% in 2011. The percentages of acupuncture users in female to those in male in every year ranged from 1.24:1 to 1.31:1 between 2002 and 2011. Residents in low urbanization areas (level 4) were less likely to receive acupuncture. Acupuncture users of different urbanized residences all increased from 2002 to 2011.

The highest percentage of acupuncture users were in the 50–59 y/o age group (9.69%), followed by the 40–49 y/o and 60–69 y/o groups (Table 2). In the old age group, including patients more than 80 y/o, the proportion of acupuncture users was only 4.9%. However, these patients had a higher average number of visits than the other groups.

**Table 2:** Age-specific prevalence of acupuncture users during 10 years from 2002–2011 in Taiwan.

Age (years)	Number of total population	Number of acupuncture users (%)	Number of acupuncture visits (visits/subject)
≥80	238059	11691 (4.91)	39390 (3.37)
70–79	478273	37573 (7.86)	118704 (3.16)
60–69	681064	61437 (9.02)	163406 (2.66)
50–59	1208056	117005 (9.69)	278441 (2.38)
40–49	1533355	142171 (9.27)	317716 (2.23)
30–39	1534700	135803 (8.85)	282865 (2.08)
20–29	1385437	120819 (8.72)	231815 (1.92)

More patients received acupuncture in TCM clinics than in hospitals (Table 3). There were 6 regional divisions considered, including Taipei and the Northern, Central, Southern, Kao-ping, and Eastern divisions. The percentages of acupuncture users were highest in the Central branch bureau and lowest in the Southern branch bureau.

**Table 3:** Service volumes of the acupuncture by facility type and region from 2002–2011 in Taiwan.

Year	Accreditation level of hospital		Location of medical institution													
			Taipei branch bureau		Northern branch bureau		Central branch bureau		Southern branch bureau		Kao-Ping branch bureau		Eastern branch bureau		Others*	
	Hospital	Clinic	N	n (%)	N	n (%)	N	n (%)	N	n (%)	N	n (%)	N	n (%)	N	n (%)
2002	2913	51193	253848	21655 (8.53)	91505	5511 (6.02)	120646	14426 (12.0)	94373	5093 (5.40)	102080	6164 (6.04)	11960	1257 (10.5)	3340	0
2003	2766	53201	255855	21491 (8.40)	91779	6065 (6.61)	121992	14459 (11.9)	95114	5557 (5.84)	103095	7073 (6.86)	11987	1322 (11.0)	3398	0
2004	3160	54960	257802	21339 (8.28)	92333	6826 (7.39)	123307	15307 (12.4)	95887	5865 (6.12)	103991	7439 (7.15)	12036	1344 (11.2)	3459	0
2005	2966	52802	260793	20183 (7.74)	93536	6810 (7.28)	125170	14445 (11.5)	96900	5769 (5.95)	105112	7200 (6.85)	12100	1361 (11.3)	3533	0
2006	2932	52137	263312	20275 (7.70)	94498	6907 (7.31)	126682	13780 (10.8)	97792	5728 (5.86)	105964	7050 (6.60)	12135	1329 (11.0)	3575	0
2007	2940	54313	265483	21389 (8.06)	95311	7279 (7.64)	128117	14135 (11.0)	98593	6171 (6.26)	106750	7050 (6.60)	12183	1229 (10.1)	3611	0
2008	2955	57574	267726	22650 (8.46)	96274	8091 (8.40)	129414	14551 (11.2)	99468	6321 (6.35)	107684	7786 (7.23)	12192	1130 (9.27)	3633	0
2009	3209	68207	269531	27109 (10.1)	96948	10051 (10.4)	130608	16379 (12.5)	100158	7098 (7.09)	108368	9291 (8.57)	12206	1488 (12.2)	3685	0
2010	3128	75607	271797	31127 (11.5)	97957	10445 (10.7)	131909	17150 (13.0)	100842	7335 (7.27)	109158	11166 (10.2)	12277	1512 (12.3)	3726	0
2011	3078	76458	273301	31717 (11.6)	98837	10169 (10.3)	133041	17188 (12.9)	101416	7087 (6.99)	109766	11013 (10.2)	12332	1567 (12.7)	3753	795 (21.2)

N: numbers of beneficiaries; n: numbers of acupuncture users.

Table 4 provides the different disease categories as reasons for receiving acupuncture treatment by different age groups. The top two disease categories leading to acupuncture visits were injury (50.6%) and diseases of the musculoskeletal system and connective tissue (41.8%).

**Table 4:** Frequency distribution of acupuncture users by disease categories and age groups from 2002–2011 in Taiwan.

	Acupuncture, n (%)	Age		
Diagnosis (ICD-9-CM range)	All	20–39 (%)	40–59 (%)	≥60 (%)
Injury and poisoning (800–999)	8160624 (50.6)	3031150 (61.7)	3630624 (51.8)	1498850 (35.7)
Diseases of the Musculoskeletal System and Connective Tissue (710–739)	6744878 (41.8)	1698062 (34.5)	2894757 (41.3)	2152059 (51.3)
Diseases of the Circulatory System (390–459)	459134 (2.85)	26925 (0.55)	135128 (1.93)	297081 (7.08)
Diseases of the Nervous System and Sense Organs (320–389)	385228 (2.39)	58516 (1.19)	170672 (2.44)	156040 (3.72)
Symptoms, Signs, and Ill-Defined Conditions (780–799)	175667 (1.09)	40811 (0.83)	83521 (1.19)	51335 (1.22)
Diseases of the Respiratory System (460–519)	42374 (0.26)	14323 (0.29)	19086 (0.27)	8965 (0.21)
Diseases of the Genitourinary System (580–629)	36374 (0.23)	10156 (0.21)	22272 (0.32)	3946 (0.09)
Mental Disorders (290–319)	35494 (0.22)	16454 (0.33)	13305 (0.19)	5735 (0.14)
Diseases of the Digestive System (520–579)	34866 (0.22)	10501 (0.21)	15581 (0.22)	8784 (0.21)
Neoplasms (140–239)	20130 (0.12)	1371 (0.03)	11565 (0.17)	7194 (0.17)
Endocrine, Nutritional, and Metabolic Diseases, and Immunity Disorders (240–279)	13359 (0.08)	2218 (0.05)	5184 (0.07)	5957 (0.14)
Diseases of the Skin and Subcutaneous Tissue (680–709)	10009 (0.06)	6003 (0.12)	3043 (0.04)	963 (0.02)
Infectious and Parasitic Diseases (001–139)	3,571 (0.02)	301 (0.01)	1941 (0.03)	1329 (0.03)
Diseases of the Blood and Blood-Forming Organs (280–289)	893 (0.01)	119 (0.00)	205 (0.00)	569 (0.01)
Complications of Pregnancy, Childbirth, and the Puerperium (630–676)	162 (0.00)	124 (0.00)	7 (0.00)	31 (0.00)

Patients suffering from stroke, including cerebral infarction and intracerebral hemorrhage, were the largest population among those receiving complex acupuncture (Table 5).

**Table 5:** The top ten diseases of patients received complex acupuncture.

Diseases	ICD-9	N = 40028 (%)
Cerebral infarction	433–434	14164 (35.4)
Intracerebral hemorrhage	431–432	8962 (22.4)
Other cerebrovascular disease	435–437	8276 (20.7)
Subarachnoid hemorrhage	430	2828 (7.07)
Cancer	140–208	1812 (4.53)
Spinal cord injury without evidence of spinal bone injury	952	953 (2.38)
Schizophrenia	295	738 (1.84)
Manic disorder	296	713 (1.78)
Fracture of vertebral column with spinal cord injury	806	497 (1.24)
Other diseases of spinal cord	336	318 (0.79)

## Discussion

Our study revealed that the one-year prevalence of acupuncture use in Taiwan increased from 7.98% in 2002 to 10.9% in 2011, with the yearly incidence rate ratio of 1.04. Women had a higher acupuncture utilization rate than men, and middle-aged groups were the most likely to receive acupuncture. Of the six regions, the percentage of one-year acupuncture users was highest in Central branch bureau. Injury and diseases of the musculoskeletal system and connective tissue were the major reason that patients received acupuncture. Patients suffering from stroke represented the largest proportion of those receiving complex acupuncture.

A previous study on the characteristics of acupuncture users in Taiwan demonstrated that the ratio of acupuncture users was fairly stable during the first few years of the establishment of the NHI program (from 6% in 1996 to 6.2% in 2002) [29]. However, our study revealed that from 2002 to 2011, acupuncture utilization increased further from 7.98% to 10.9%, which was consistent with the increasing trend in overall TCM users in Taiwan (from 26.59% in 2000 to 28.66% in 2010) [3]. The percentage of acupuncture usage in Taiwan was higher than that in Japan (6.7% in 2006) [4], Australia (3.4% in 2011) [30], the U.S.A. (1.5% in 2007) [9], the UK (1.6% in 2004) [31] and South Korea (7.4%) [6]. Although acupuncture has become more popular in Asian countries, the proportion of Taiwanese acupuncture users was still lower than the proportion of overall TCM users in Taiwan. This might be due to the wide acceptance of Chinese herbal medicine in Taiwan. TCM theories such as Yin and Yang as well as Chinese herbal medicine are part of life and Chinese culture. For example, several herbs are commonly used to promote health in Taiwanese communities [32]. In addition, Chinese herbal medicine is also reimbursed by NHI policy and is thus easily affordable by the general public.

Acupuncture users were mainly enrollees in the middle-aged groups, which is similar to the findings of previous reports on acupuncture users in Taiwan [5], Australia [8] and the U.S.A. [33]. In accordance with previous studies in Taiwan [5], Japan [4], the UK [34] and Australia [30], our study revealed a female predominance among acupuncture users. Residents in highly urbanized areas usually utilized TCM more than those in less urbanized areas [35].

The most common diseases among patients receiving acupuncture treatment in Taiwan were injury and disorders of the musculoskeletal system and connective tissue, which is similar to the results in Japan [4] and Australia [8]. According to TCM theory, these disorders are considered to represent Qi stagnation and blood stasis in terms of TCM diagnoses. Acupuncture is usually considered to move qi and remove blood stasis quicker than Chinese herbal medicine [36]. Previous studies among osteoarthritis [37] and fibromyalgia [38] patients also provided substantial evidence supporting the beneficial effects of acupuncture in these disorders.

Patients with circulatory disorders, including cardiovascular disease and cerebrovascular disorders, also received acupuncture. The proportion of acupuncture users among stroke patients in Taiwan was 17% in 2008 [26]. A retrospective cohort study revealed that acupuncture lowered the rate of recurrent stroke in patients with ischemic stroke [39]. Furthermore, a previous study found that ischemic stroke patients experienced increased cerebral blood flow when they received acupuncture treatment [40]. The NHIA launched a Pilot Scheme for Health Policy in Stroke Adjuvant Acupuncture Therapy (HPSAAT) in 2006 to support the health care of stroke patients [41]. In addition to outpatient clinical consultations, the inpatient treatment of stroke patients in acute and subacute stages with acupuncture has been reimbursed by the NHIA since 2006. The implementation of this pilot scheme in the NHI policy has also promoted the utilization and integration of acupuncture treatment in stroke patients.

Cancer patients often seek CAM to ameliorate symptoms induced by cancer or the side effects of cancer treatment [24]. Notably, cancer, which was the tenth leading disease category among acupuncture visits in our study, was not in the top 10 disease categories among acupuncture visits in the previous study in Taiwan [5]. The number of cancer patients has increased in recent years, and the acceptability of TCM theory has also been growing. Acupuncture research in the field of oncology has also increased [42]. Current evidence has found that acupuncture is effective in treating the symptoms associated with cancer and cancer treatment. For example, cancer pain was shown to be attenuated through auricular acupuncture [43]. Acupuncture significantly improved joint pain in postmenopausal women with aromatase inhibitor-induced arthralgia [44]. Furthermore, fatigue, anxiety and depression in women with aromatase inhibitor-induced arthralgia were also improved by acupuncture [45]. Complementary TCM treatment approaches for hospitalized cancer patients, including acupuncture, are also covered by the NHI.

There were several limitations to our study. First, detailed information about acupuncture, including the selected acupoints, manipulation and needle retention time, were not provided in this dataset. The detail information could be only provided in the electronic medical records in hospitals but not in the dataset for study from NHIRD. We could only analyze the utilization rate in different population and different disease categories. Another limitation was that the treatment codes recorded in the NHIRD only included manual acupuncture, electroacupuncture and complex acupuncture. Auricular acupuncture, scalp acupuncture and moxibustion were all recorded under the same treatment codes as manual acupuncture. Acupressure may have been recorded as orthopedic manipulation. However, this study still provided the most up-to-date information about the seeking of acupuncture treatment, the associations of demographic data and the trends in acupuncture utilization in Taiwan. The large sample size provided by the NHIRD also minimized selection bias.

## Conclusion

This study provides the most up-to-date report on acupuncture utilization among adults in Taiwan. The major characteristics of acupuncture users included being middle-aged, female and a resident of a highly urbanized area and suffering from injury or disorders of the musculoskeletal system and connective tissue. Our findings may provide useful information for clinical practice and acupuncture research as well as for health policy decision making.
